# CRISPR-Cas12a-based fluorescent and visual assays for universal detection and clade discrimination of mpox virus

**DOI:** 10.1128/spectrum.00105-26

**Published:** 2026-06-22

**Authors:** Yingqing Mao, Xinyu Fei, Xijing Yang, Cheng Hu, Changqiang Zhu, Junhu Wang, Yixin Ge, Chuchu Ye, Sunjie Yang, Peng Cheng, Yuexi Li, Xiaobing He, Zhiliang Hu, Yong Qi

**Affiliations:** 1 Huadong Research Institute for Medicine and Biotechniques, Nanjing, China; 2 School of Life Sciences, Xuzhou Medical University 38044 , Xuzhou, China; 3 School of Engineering, China Pharmaceutical University 630772, Nanjing, China; 4 The 907th Hospital of Chinese PLA, Nanping, China; 5 Huangshan Center for Disease Control and Prevention, Huangshan, China; 6 State Key Laboratory for Animal Disease Control and Prevention, Lanzhou Veterinary Research Institute, Chinese Academy of Agricultural Sciences 111658 , Lanzhou, China; 7 Department of Infectious Disease, The Second Hospital of Nanjing, Nanjing University of Chinese Medicine 66478, Nanjing, China; Children's National Hospital, George Washington University, Washington, DC, USA

**Keywords:** mpox virus, CRISPR-Cas12a, recombinase-aided amplification, G-quadruplex, nucleic acid detection, diagnosis

## Abstract

The global mpox outbreak has highlighted critical gaps in diagnostic capabilities, particularly the need for methods that can distinguish between the high-fatality Clade I and more transmissible Clade II of the mpox virus (MPXV). Current PCR-based approaches remain reliant on laboratory infrastructure, limiting their use in resource-limited settings. There is an urgent need for a versatile diagnostic platform that can provide both accurate clade discrimination and flexible deployment across diverse healthcare environments. We developed a dual-mode detection platform by integrating recombinase-aided amplification with Clustered Regularly Interspaced Short Palindromic Repeats-Cas12a technology, creating two distinct assays: a universal assay targeting OPG034, and a Clade I-discriminatory assay targeting OPG033. The platform achieved detection sensitivities of 1 copy/reaction for both fluorescence and visual readouts with OPG034, and 10 copies (fluorescence) and 1 copy/reaction (visual) with OPG033. Both assays demonstrated high specificity, successfully distinguishing MPXV from related orthopoxviruses and common viruses. Clinical validation using 23 Clade II samples and 10 healthy controls showed that, relative to quantitative PCR (qPCR) (cycle threshold ≤37), the OPG034 fluorescence assay detected all 13 qPCR-positive samples and 2 additional positives (100% sensitivity, 80.0% specificity), while the visual assay detected 12 of 13 positives (92.3% sensitivity, 100% specificity). For Clade I-specific detection, both OPG033 fluorescence and visual assays showed 100% specificity in Clade II samples (23/23). While the clade-discriminatory capability was established through sequence-specific design, further evaluation with authentic Clade I clinical specimens is warranted to confirm typing performance. The dual-mode design provides flexibility for both laboratory and field use, advancing mpox surveillance and outbreak response.

**IMPORTANCE:**

Mpox is a significant zoonosis. Accurate discrimination between its highly lethal Clade I and more transmissible Clade II is critical for clinical management and outbreak control, yet current methods primarily enable only general detection. To address this, we identified novel genetic markers (OPG034 for universal detection and OPG033 for Clade I specificity) and developed a dual-mode detection platform integrating Clustered Regularly Interspaced Short Palindromic Repeats-Cas12a with recombinase-aided amplification. Its key advantage is providing two result readouts: a sensitive fluorescence mode for laboratories and an instrument-free visual colorimetric mode for field use. The demonstrated excellent performance on clinical samples confirms that this platform meets the precision requirements of clinical laboratories while remaining suitable for resource-limited settings like field clinics. Thus, it offers a flexible and practical tool for enhancing mpox surveillance and control globally, particularly in regions with constrained medical resources.

## INTRODUCTION

Mpox is a viral zoonosis caused by the mpox virus (MPXV). MPXV belongs to the *Orthopoxvirus* genus of the Poxviridae family, which also includes related viruses such as variola virus (VARV) and vaccinia virus (VACV) (1). MPXV transmission occurs through multiple routes, including close contact with or bites from infected animals, respiratory droplets, contact with skin lesions, vertical transmission, and sexual transmission (2). The incubation period typically ranges from 5 to 21 days, with common clinical manifestations such as fever, headache, myalgia, lymphadenopathy, and a characteristic rash (3, 4). Since May 2022, global mpox outbreaks have been reported in many regions worldwide. As of August 2024, the cumulative number of confirmed cases has exceeded 100,000 (5, 6). The World Health Organization has declared the mpox outbreak a Public Health Emergency of International Concern on two separate occasions (7). Experience from the COVID-19 pandemic underscores the importance of accurately identifying high-risk populations for effective epidemic control. However, many regions still lack large-scale testing capacity, leading to delayed public health responses and limited containment efficacy (8). Therefore, the development of accurate, rapid, and low-cost detection methods is of great significance for MPXV surveillance and control (9).

MPXV is divided into two major clades: Clade I (Congo Basin clade) and Clade II (West African clade), which exhibit notable differences in epidemiological characteristics (10). From an epidemiological perspective, Clade I is predominantly endemic to Central Africa, particularly the Democratic Republic of Congo, and is characterized by higher pathogenicity. In contrast, Clade II is mainly distributed in West Africa, with subclade IIb serving as the primary variant responsible for the 2022–2023 global mpox outbreak, establishing sustained human-to-human transmission across more than 100 countries (9, 10). In 2024, a novel subclade Ib emerged in Central Africa and subsequently spread to neighboring countries and other continents, posing an emerging threat to global public health. Clade I is associated with higher pathogenicity and a case fatality rate of up to 12%, whereas Clade II, though less pathogenic, demonstrates higher transmissibility (11, 12). Thus, rapid and accurate clade identification is crucial for guiding clinical management and formulating public health strategies. However, discriminating between the two clades is challenging due to their minimal genomic sequence divergence (approximately 0.5%) (1) and high homology with other orthopoxviruses (such as VARV and cowpox virus [CPV]) in conserved regions (e.g., D6R, E9L). These factors complicate conventional PCR design and often lead to cross-reactivity or misclassification (13–16). Most current assays only support universal MPXV detection and lack clade-discriminatory capability (9).

Single nucleotide polymorphisms (SNPs) offer a means for specific identification, yet conventional methods like quantitative PCR (qPCR) and isothermal amplification exhibit limited SNP discrimination. In contrast, the Clustered Regularly Interspaced Short Palindromic Repeats (CRISPR) technology shows high specificity for SNP recognition and has been successfully applied to MPXV detection (17–22). The system employs Cas proteins (e.g., Cas12, Cas13) guided by crRNA to recognize targets, activating trans-cleavage that degrades reporter molecules to generate detectable signals (23). Although less sensitive than qPCR, incorporating pre-amplification (e.g., recombinase-aided amplification [RAA]) enhances sensitivity to the attomolar level (23). CRISPR assays are also instrument-flexible and support multiple readouts, such as fluorescence and lateral flow strips (24–27).

Recent advances in CRISPR-based diagnostics for MPXV have demonstrated considerable potential for rapid and accurate detection. Current strategies predominantly target conserved viral genes, including F3L and A27L, achieving high analytical sensitivity through integration with isothermal pre-amplification techniques such as RAA, recombinase polymerase amplification (RPA), loop-mediated isothermal amplification (LAMP), and multiple cross displacement amplification (MIRA) (20, 26, 28, 29). For example, fluorescence-based platforms combining RAA or RPA with CRISPR/Cas12a or Cas12b systems have reported limits of detection as low as 1–10 copies/μL (26, 28). To facilitate point-of-care testing, several studies have incorporated lateral flow assay strips for visual readout (26, 29) or developed one-pot reaction systems to minimize the risk of nucleic acid contamination (20). Despite these advancements, most existing methods are designed for universal MPXV detection and exhibit limited capacity to differentiate between the epidemiologically distinct Clade I and Clade II lineages (20, 26, 29). Moreover, while lateral flow strips enable instrument-free visual detection, their sensitivity is generally lower than that of fluorescence-based approaches (26). Therefore, the development of a simplified diagnostic platform that simultaneously enables high-sensitivity clade discrimination and robust, equipment-free visual detection remains a critical unmet need in MPXV diagnostics.

DNAzymes are functional DNA molecules with catalytic activity. For example, G-quadruplex structures can exhibit peroxidase-like activity upon binding to hemin, catalyzing the colorimetric reaction of 2,2′-azinobis (3-ethylbenzothiazoline-6-sulfonic acid) (ABTS) in the presence of hydrogen peroxide (H_2_O_2_), thereby enabling visual or instrumental interpretation (30, 31). By leveraging the trans-cleavage activity of Cas12a toward ssDNA, the CRISPR system can be integrated with a DNAzyme-mediated colorimetric system to establish an instrument-free visual detection method. This approach is low-cost and simple to operate, making it particularly suitable for field screening in resource-limited settings (32).

Here, we established a dual-mode detection platform combining RAA pre-amplification and CRISPR-Cas12a. It supports both fluorescent readouts for laboratory use and DNAzyme-based colorimetry for field screening, enabling universal MPXV detection and clade identification to support clinical and public health needs.

## MATERIALS AND METHODS

### Materials and reagents

The RAA nucleic acid amplification kit (Basic Version) was purchased from Jiangsu Qitian Gene Biotechnology Co., Ltd. (Wuxi, China). LbCas12a nuclease was obtained from GenScript (Nanjing, China). NEBuffer r2.1 was acquired from New England Biolabs (Beijing, China). Hemin chloride was sourced from Solarbio (Beijing, China). The EL-ABTS chromogenic kit and TMB chromogenic solution (for ELISA) were purchased from Sangon Biotech (Shanghai, China). H_2_O_2_ was supplied by Hanno (Huainan, China). The Monkeypox Virus Nucleic Acid Detection Kit was purchased from Da’an Gene Co., Ltd. (Guangzhou, China). QIAamp DNA Blood & Tissue Kit was acquired from QIAGEN (Hilden, Germany). All oligonucleotides, including plasmids, crRNAs, primers, reporters, and G4 single-stranded DNA, were chemically synthesized by Sangon Biotech (Shanghai, China). The sequences of all DNA and RNA oligonucleotides used in this study are listed in Table 1. All other chemical reagents were also obtained from Sangon Biotech.

**TABLE 1:** Oligonucleotide sequences employed in this work

Names	Sequences (5′−3′)
OPG034-crRNA1	UAAUUUCUACUAAGUGUAGAUGGCGUAGGCGUUAGUUUAGUUUC
OPG034-F1	TTTTTTTGAACAATGGACCTAGTGGTATAT
OPG034-F2	GGTATATCTTGTTCTATGTATCTAAAATAA
OPG034-F3	TAGTTTAATATCCGCAGTCATCTTGTCTAG
OPG034-R1	GCTATTATATTTTTGGTGTTAGATAAAGAC
OPG034-R2	CGGATGATTGTAATAAAATATTAGCTATTA
OPG034-R3	TTAGTTAGTGAGATCGTAGAAGAATATCCG
OPG033-crRNA	UAAUUUCUACUAAGUGUAGAUUGACUAAUGCUAUAUGCAAUGC
OPG033-crRNA(−1)	UAAUUUCUACUAAGUGUAGAUUGACUAAUGCUAAAUGCAAUGC
OPG033-crRNA(−2)	UAAUUUCUACUAAGUGUAGAUUGACUAAUGCUUUAUGCAAUGC
OPG033-crRNA(−3)	UAAUUUCUACUAAGUGUAGAUUGACUAAUGCAAUAUGCAAUGC
OPG033-crRNA(+1)	UAAUUUCUACUAAGUGUAGAUUGUCUAAUGCUAUAUGCAAUGC
OPG033-crRNA(+2)	UAAUUUCUACUAAGUGUAGAUUCACUAAUGCUAUAUGCAAUGC
OPG033-crRNA(+3)	UAAUUUCUACUAAGUGUAGAUAGACUAAUGCUAUAUGCAAUGC
OPG033-F1	GTTCTAGGAGAGTAATGGGTTATTGTGGA
OPG033-F2	TCTGTATTACCGACTATCGTCCAATAT
OPG033-R1	TATAGCATTGTTGGGATTAACTATATC
OPG033-R2	ATTTTACATCCCAAGTTTATGACTAATG
OPG033-R3	TCCAAACGATGTAGATATCATCAACATT
Fluorescence reporter	FAM-TTTTTTTTTTTT-BHQ1
G2c	TTGGGTAGGGC
G2d	GGGTTGGGAAA
G3a	TGGGTAGGGCGGGAAA
CatG4	TGGGTAGGGCGGGTTGGGAAA

### Screening of MPXV targets for universal and clade-specific detection

To identify target sequences for the universal detection of MPXV, genome sequences of MPXV, VARV, VACV, camelpox virus (CMLV), and CPV were downloaded from the National Center for Biotechnology Information database (http://www.ncbi.nlm.nih.gov). Whole-genome alignment was performed using MAUVE software (version 20150226, Darling Lab, University of Technology Sydney) (33) to screen for MPXV-specific sequences longer than 300 bp. Phylogenetic analysis of candidate sequences was conducted using the Nextclade database (https://clades.nextstrain.org/) to confirm their affiliation with MPXV. Finally, the BLAST program (http://blast.ncbi.nlm.nih.gov/) was used to align candidate sequences against all available MPXV and non-MPXV viral gene sequences to verify the specificity.

For clade-specific targets, genomes of MPXV Clade I and Clade II were compared using MAUVE to identify unique sequence fragments present only in Clade I. Phylogenetic analysis was performed using Nextclade to confirm the clade specificity of candidate sequences. Subsequently, candidate sequences were subjected to BLAST analysis against all the available non-MPXV viral genomes to confirm their specificity for MPXV.

The selected universal detection target (OPG034) and Clade I-specific target (OPG033) sequences were chemically synthesized by Sangon Biotech and cloned into the pUC57 plasmid to generate positive control plasmids for subsequent assay development.

### Development of a Cas12a-based detection method

Multiple crRNAs were designed for each selected target sequence. Each crRNA consisted of a 21-nt universal sequence (5′-UAAUUUCUACUAAGUGUAGAU-3′) (34) and a 19–24 nt sequence complementary to the target region. The performance of candidate crRNAs was evaluated using a CRISPR-Cas12a fluorescence detection system as previously described (35, 36). Briefly, each 20 µL reaction mixture contained 1 × NEB Buffer 2.1, 20 nM LbCas12a, 100 nM crRNA, 50 nM fluorescent reporter, and 10^10^ copies of a positive control plasmid. Reactions were incubated at 37°C in an F1620 fluorescence microplate reader (Qitian Gene Biotechnology Co., Ltd.) for real-time fluorescence monitoring. The pUC57 empty plasmid was used as a negative control template. Fluorescence kinetics were recorded, and the slope of the signal increase was calculated to evaluate the detection efficiency. The crRNA showing the highest positive-to-negative slope ratio was selected as the optimal candidate.

### RAA detection development

Following the selection of the optimal crRNA, multiple forward and reverse RAA primers were designed using Primer Premier 5 software, targeting regions within approximately 200 bp upstream and downstream of the crRNA binding site, respectively. The amplification efficiencies of all primer combinations were evaluated using a commercial RAA nucleic acid amplification kit (Basic Version) according to the manufacturer’s instructions. Each 45 µL RAA reaction mixture contained 2 µL each of forward and reverse primers (10 µM), 1 µL of template (1,000 copies/µL), 25 µL of reaction buffer, and 15 µL of ddH_2_O. The mixture was added to a tube containing lyophilized RAA enzyme, and the reaction was initiated by adding 5 µL of magnesium acetate. Amplification was carried out at 37°C for 20 min in a B6100 vibrating mixer (Qitian Gene Biotechnology Co., Ltd.). After amplification, 2 µL of the product was used as the template in the CRISPR fluorescence detection system. The primer set yielding the strongest fluorescence signal and shortest time-to-peak was selected as the optimal amplification primer combination.

### Establishment of the CRISPR/Cas12a-DNAzyme detection system

Multiple G-rich ssDNA sequences with different compositions were evaluated for their color development capability. The evaluation was performed by preparing 20 µL of G-rich ssDNA solutions with varying concentrations, followed by the addition of 1 µL of hemin chloride (50 µM) and 2 µL of potassium chloride (500 µM). The mixtures were incubated at 37°C for 10 min, after which 50 µL of ABTS chromogenic reagent (EL-ABTS chromogenic kit) was added to develop color. Color changes were assessed visually, and absorbance at 405 nm was measured using a Spark microplate reader (TECAN, Switzerland). The G-rich ssDNA demonstrating the most significant color response was selected for subsequent experiments.

The optimal G-rich ssDNA was then used to replace the fluorescent reporter in the established CRISPR/Cas12a detection system. In the optimized system, 10^10^ copies of the pUC57-OPG034 plasmid were used as templates and incubated at 37°C for 1 h. Following incubation, hemin chloride, potassium chloride, and ABTS chromogenic reagent were added. Color development was monitored visually at room temperature, and absorbance values were recorded.

### Establishment and evaluation of the RAA-CRISPR/Cas12a fluorescence and DNAzyme methods

The optimized RAA system was integrated with the CRISPR/Cas12a fluorescence detection system and the CRISPR/Cas12a-DNAzyme colorimetric system to establish two distinct detection platforms: a fluorescence-based method and a naked-eye visual method. The procedure was as follows: target fragments were first amplified using the RAA nucleic acid amplification kit (Basic Version). Then, 2 μL of the RAA product was transferred to the CRISPR fluorescence detection system for fluorescence signal measurement, while 0.25 μL of the product was added to the CRISPR/Cas12a-DNAzyme system for colorimetric analysis.

The limits of detection (LOD) for both methods were assessed using serial dilutions of a positive plasmid. The plasmid was diluted with elution buffer to concentrations of 1,000, 100, 10, and 1 copy/μL, and each dilution was subjected to amplification and detection. For each dilution, the plasmid templates were first amplified via RAA, and the resulting products were then used for CRISPR/Cas12a-based detection. The LOD was defined as the lowest template concentration at which a positive signal could be consistently detected. The pUC57 empty plasmid was included as a negative control in all experiments.

### Clinical sample collection and nucleic acid extraction

Nine plasma samples were collected from laboratory-confirmed MPXV Clade II-infected patients at Nanjing Second Hospital, and fourteen confirmed MPXV Clade II rash fluid swab samples were obtained from the Jiangsu Provincial Center for Disease Control and Prevention. The samples were collected from patients at various stages of infection; some patients presented with typical symptoms, while others had mild symptoms and were likely in the recovery period. For specificity evaluation, the following clinical samples were included: one cytomegalovirus (CMV) serum sample, one human papillomavirus type 3 (HPV-3) serum sample, and one Epstein-Barr virus (EBV) serum sample provided by the Huangshan City Center for Disease Control and Prevention; three lesion swab samples from varicella-zoster virus (VZV)-infected patients were provided by the 907th Hospital of the PLA. Genomic DNA of the vaccinia Tiantan strain was maintained in our laboratory. Blood samples from 10 healthy donors were collected from a participating hospital as negative controls.

DNA extraction was performed using the QIAamp DNA Blood & Tissue Kit according to the manufacturer’s instructions. The presence of target viruses was confirmed in advance using corresponding qPCR assays.

### Real-time fluorescence PCR detection of MPXV

Real-time fluorescence PCR was conducted using a commercial Monkeypox Virus Nucleic Acid Detection Kit (Fluorescence PCR Method). The reaction mixture was prepared as follows: 17 μL of MPXV PCR Reaction Mix A and 3 μL of MPXV PCR Reaction Mix B were combined with 10 μL of the extracted viral DNA template. The amplification protocol consisted of 2 min at 50°C and 5 min at 95°C, followed by 45 cycles of 5 s at 95°C and 35 s at 60°C. Fluorescence signals were recorded using an Applied Biosystems QuantStudio 3 Real-Time PCR System (Thermo Fisher Scientific). In accordance with the Technical Guidelines for Monkeypox Control issued by the Chinese National Health Commission (37), a cycle threshold (Ct) value ≤37 was defined as positive, while a Ct value >37 was considered negative.

### Data analysis

Statistical analysis was conducted using GraphPad Prism version 8.3.0 (GraphPad Software, San Diego, CA, USA). Data from sensitivity, specificity, and primer/DNAzyme screening experiments are presented as the mean ± standard deviation (SD). For comparisons involving more than two groups, one-way analysis of variance (ANOVA) followed by Dunnett’s multiple comparison test (when comparing multiple groups to a single control) was applied, as indicated in the figure legends. For experiments analyzing the effect of two independent variables, two-way ANOVA with Bonferroni’s *post hoc* test was used. Statistical significance was defined as a *P*-value of less than 0.05 (*P* < 0.05). The sample sizes and specific statistical tests used for each experiment (e.g., biological replicates for optimization, technical replicates for validation) are detailed in the respective figure legends.

## RESULTS

### Design of the study

**Fig 1:**
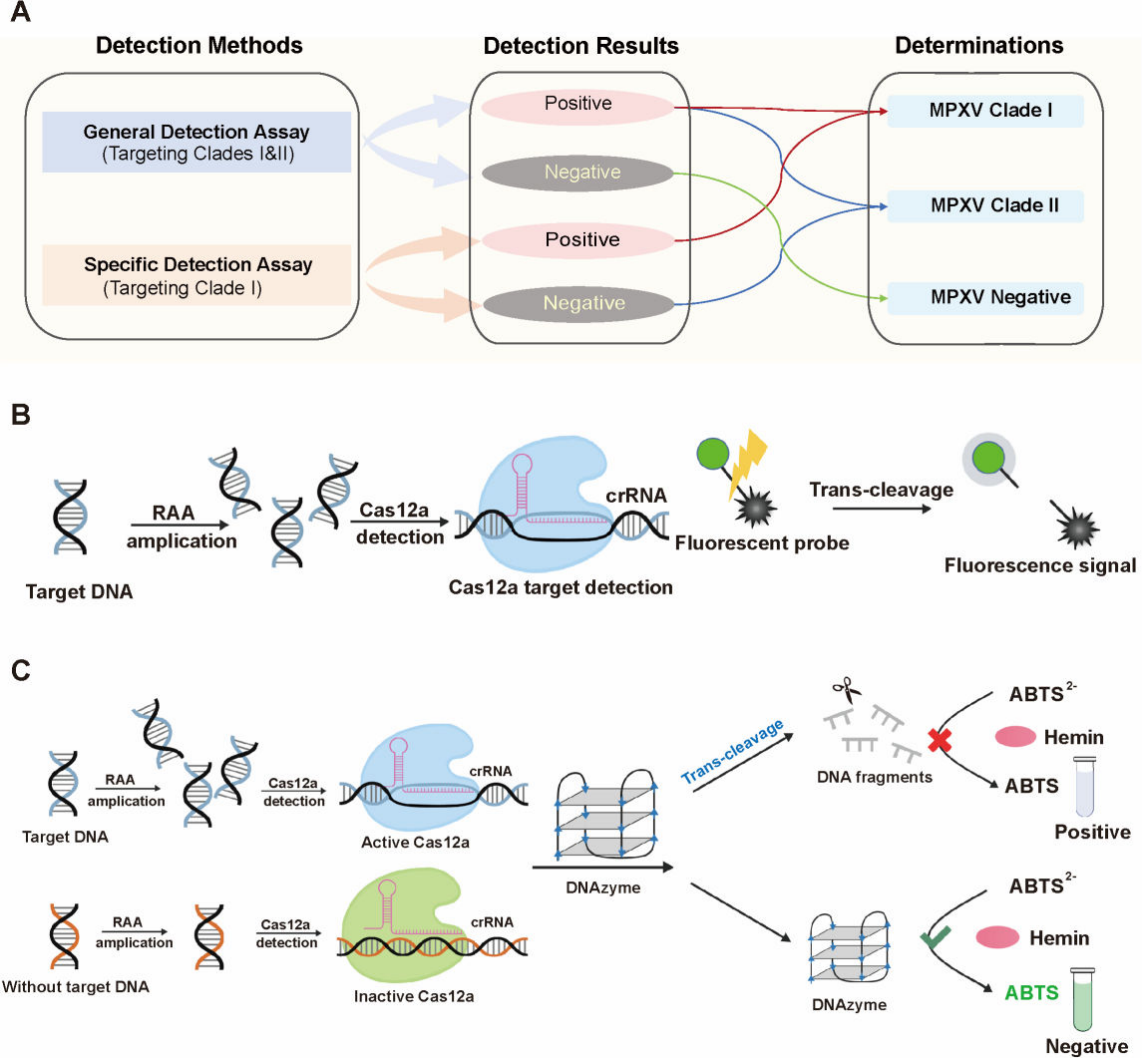
Design of the study. (**A**) The MPXV clade discrimination strategy of this study; (**B**) the reaction principle of the fluorescence detection method; (**C**) the reaction principle of the visual detection method.

We developed two CRISPR-based assays for MPXV: a pan-clade assay (detecting both Clade I and II) and a Clade I-specific assay. As shown in Fig. 1A, the combined use of these two assays enables clear discrimination among samples positive for Clade I, positive for Clade II, or negative for MPXV. Both assays are compatible with two readout formats: fluorescence and visual, naked-eye detection.

For the fluorescence-based assay, we used recombinase-aided amplification coupled with CRISPR/Cas12a-fluorescence (RAA-CRISPR/Cas12a-fluorescence, RCCF) (Fig. 1B) (35, 36). In this system, Cas12a complexed with crRNA binds target DNA following RAA, activating trans-cleavage that cleaves a quenched fluorescent reporter for signal detection.

For visual detection, we implemented an RAA-CRISPR/Cas12a-DNAzyme (RCCD) method (Fig. 1C) (35, 36). Target-activated Cas12a cleaves the DNAzyme, inhibiting its peroxidase activity and preventing ABTS²⁻ oxidation. Thus, the solution remains colorless for positive samples, while a blue-green color develops in the absence of target.

### Screening of specific genes and crRNAs

**Fig 2:**
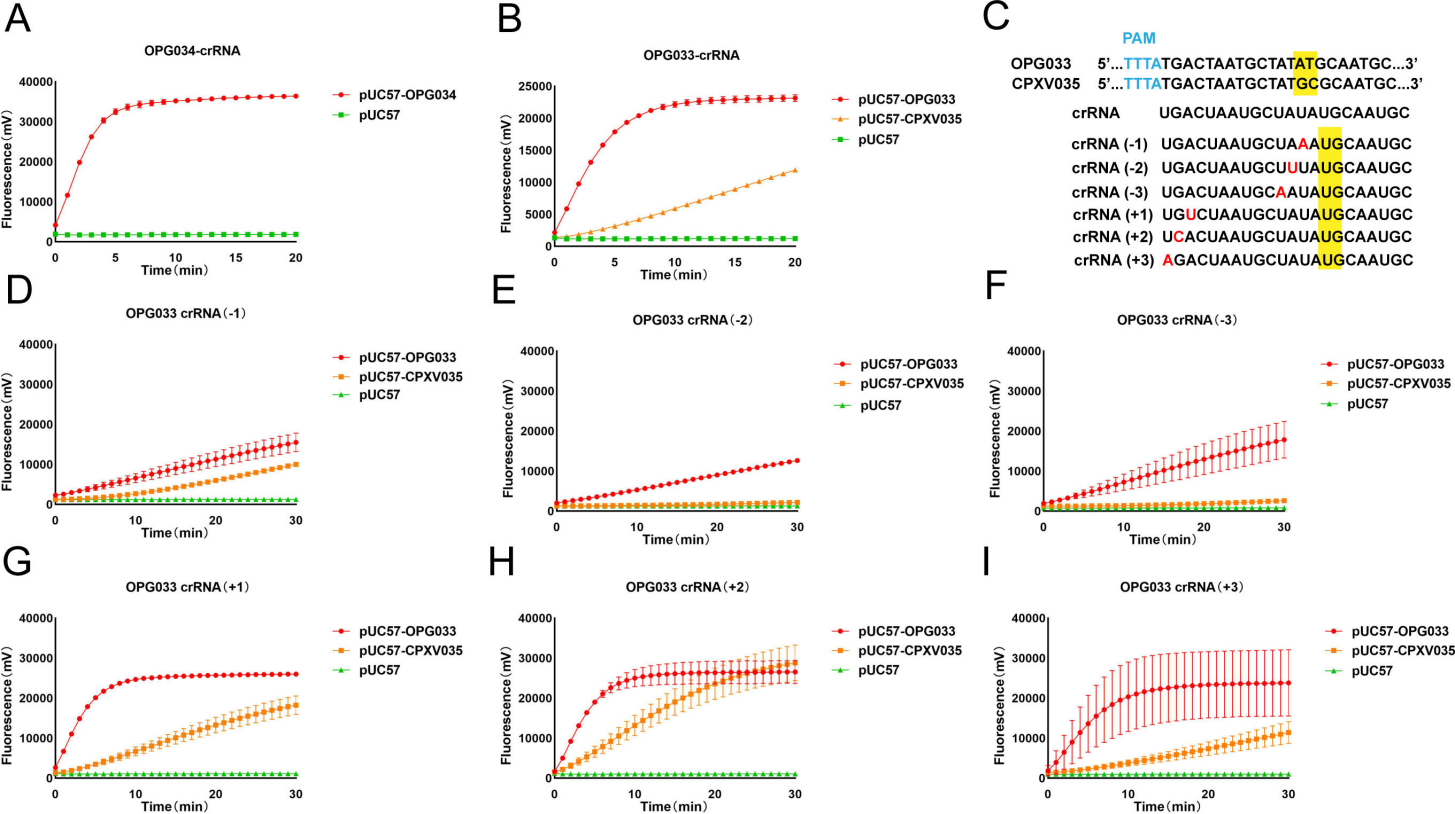
Screening and optimization of crRNAs for CRISPR-based detection. (**A**) Detection of the pUC57-OPG034 plasmid (containing the target sequence) and empty vector pUC57 (negative control) using the CRISPR/Cas12a fluorescence system. (**B**) Fluorescence detection of the pUC57-OPG033 and pUC57-CPXV035 plasmids using an OPG033-specific crRNA. (**C**) Schematic of the mismatch design for OPG033-targeting crRNAs. Six crRNA variants were constructed: three with single-base mismatches at PAM-adjacent positions +1 to +3, and three with mismatches at upstream positions −3 to −1 relative to two consecutive divergent bases between OPG033 and CPXV035. (**D–**I) Fluorescence kinetics of pUC57-OPG033 and pUC57-CPXV035 plasmids detected by the six mismatch-containing crRNAs using the CRISPR/Cas12a system. Data are presented as mean ± standard deviation from two technical replicates.

Using MAUVE software, we identified a 301 bp sequence named OPG034, located at positions 19595–19895 in the MPXV genome (GenBank accession no. NC_063383.1). Phylogenetic analysis via the Nextclade database (https://clades.nextstrain.org/) confirmed that this sequence is present in both clades of MPXV (Fig. S1 and S2). BLAST alignment confirmed that the OPG034 sequence is present in all 5,000 MPXV genomes analyzed (Fig. S3). Notably, this sequence was also detected in some VACV, VARV, and CMLV genomes (Fig. S4). However, nucleotide variations of varying lengths were observed within the region spanning positions 140–160 (minimum difference: 6 bp, see Fig. S5). A specific crRNA was designed targeting this variable region to achieve specific detection of MPXV. Experimental results confirmed that a significant fluorescence increase was observed only in reactions using the pUC57-OPG034 plasmid as templates (Fig. 2A).

Similarly, using MAUVE software, we identified a 336 bp sequence designated OPG033, located at positions 20570–20905 of the MPXV genome (GenBank accession no. NC_003310). Phylogenetic analysis with Nextclade confirmed that this sequence is exclusively present in MPXV Clade I and absent in Clade II (Fig. S6 and S7). Further BLAST analysis revealed that OPG033 is also present in some VACV, VARV, and CMLV genomes (Fig. S8), but exhibits nucleotide variations around position 167. The minimal sequence divergence was observed compared to CPXV035, showing a difference of two consecutive bases (Fig. S9).

Initially, we designed a specific crRNA targeting this two-base differential site for OPG033 recognition. However, experimental results demonstrated that both pUC57-OPG033 and pUC57-CPXV035 plasmids produced strong fluorescence signals (Fig. 2B), indicating that this crRNA failed to achieve specific detection of OPG033.

To enhance recognition specificity, we systematically engineered base mismatches in the crRNA sequences. Three single-base mismatch crRNAs were designed at positions + 1 to +3 relative to the PAM sequence. Additionally, three more mismatched variants were created by introducing substitutions at positions −3 to −1 upstream of the two consecutive differential bases between OPG033 and CPXV035 (Fig. 2C). In total, six mismatched crRNA variants were constructed and screened.

The results showed that OPG033-crRNA(−2) and OPG033-crRNA(−3) specifically recognized the pUC57-OPG033 plasmid without cross-reacting with pUC57-CPXV035 (Fig. 2E and F). The remaining four crRNAs exhibited varying degrees of non-specific recognition. Based on these findings, we successfully identified optimal crRNAs suitable for both universal and Clade I-specific detection of MPXV.

### RAA assay establishment

To enhance detection sensitivity, we established an RAA method for pre-amplification of target sequences. Multiple primer pairs spanning approximately 200 bp upstream and downstream of the crRNA binding site were designed and systematically evaluated using the CRISPR fluorescence detection system.

**Fig 3:**
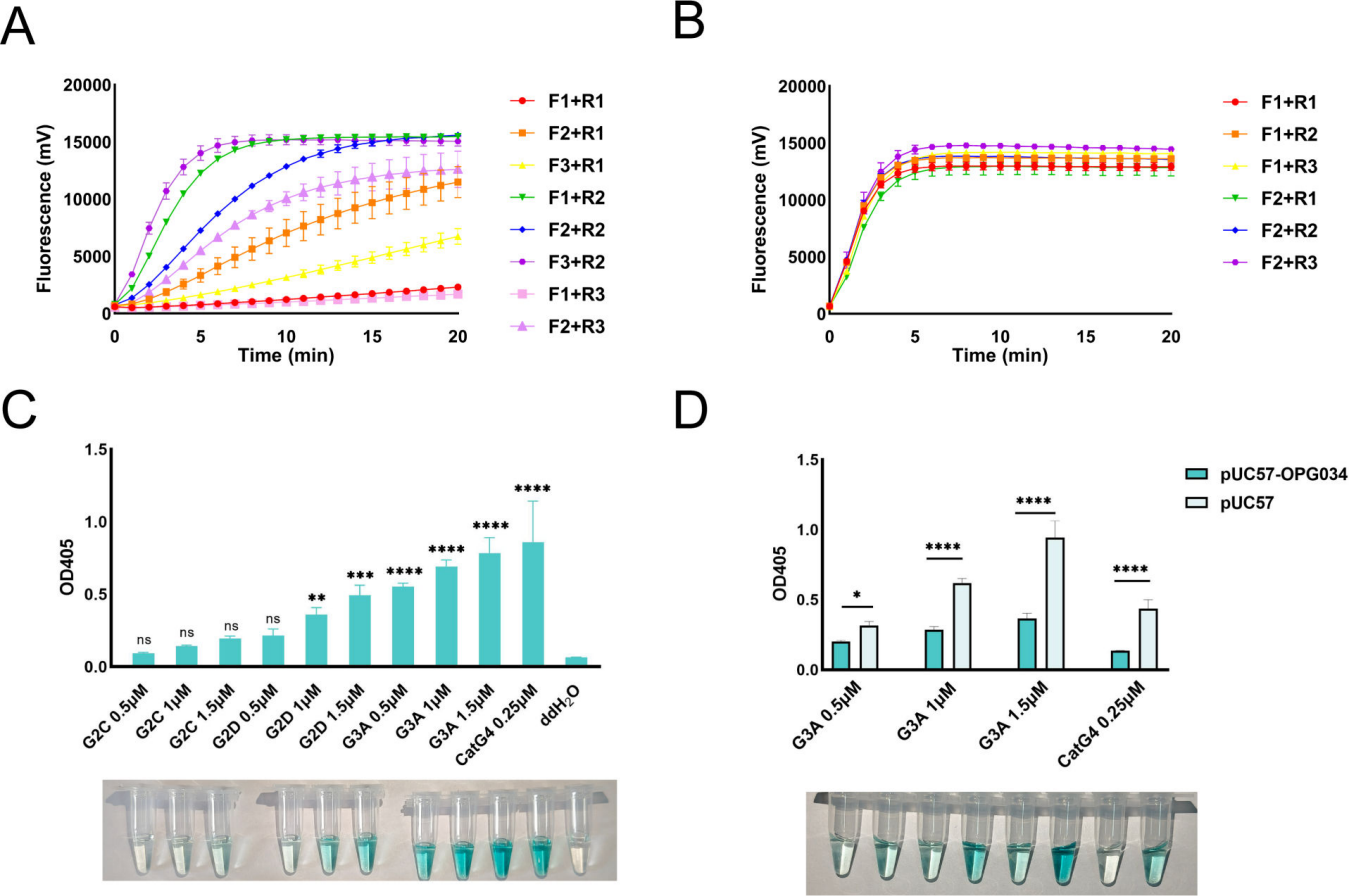
Screening of RAA primer pairs and G-rich ssDNA. (**A**) Real-time fluorescence curves of CRISPR detection for amplification products from eight RAA primer pairs targeting OPG034. (**B**) Real-time fluorescence curves of CRISPR detection for amplification products from six RAA primer pairs targeting OPG033. (**C**) Color development catalyzed by different G-rich ssDNA sequences in the ABTS²⁻-H₂O₂ chromogenic system, with ddH₂O as the negative control. (**D**) Colorimetric performance evaluation of different G-rich ssDNA sequences in the CRISPR/Cas12a-DNAzyme detection system. All experimental data are presented as mean ± SD from two technical replicates. Statistical analysis was performed using one-way ANOVA with Dunnett’s test for Fig. 2C, and two-way ANOVA with Bonferroni’s test for Fig. 2D. Significance levels: ns, not significant; *, *P* < 0.05; **, *P* < 0.01; ***, *P* < 0.001; ****, *P* < 0.0001.

For OPG034 amplification, the primer pair F3/R2 demonstrated the shortest time-to-peak and the strongest fluorescence intensity, indicating superior amplification performance, and it was therefore selected as the optimal primer pair for OPG034 (Fig. 3A). All six primer pairs tested for OPG033 showed strong fluorescence signals and short detection times, suggesting robust amplification capacity (Fig. 3B). From these, the F2/R3 primer pair was chosen for subsequent experiments.

### Selection and optimization of G-rich ssDNA in the CRISPR/Cas12a-DNAzyme system

Previous studies have reported that G-triplex structures, similar to G-quadruplexes, can bind hemin to form DNAzymes with peroxidase-like activity, catalyzing chromogenic reactions (38, 39). In addition, our previous experiments indicated that Cas12a cleaves G-rich sequences less efficiently, likely due to the formation of stable secondary structures that hinder enzymatic cleavage. To address this, we designed four G-rich sequences comprising varying numbers of tandem G-rich repeats: G2C and G2D each contain two tandem G-repeat blocks, G3A contains three, and CatG4 contains four such blocks.

The catalytic activities of these sequences were evaluated using an ABTS²⁻-H₂O₂ chromogenic assay. G3A and CatG4 showed pronounced color development ability, producing a blue color whose intensity was concentration-dependent. G2D exhibited moderate activity, while G2C showed almost no color change (Fig. 3C). When tested in the CRISPR/Cas12a-DNAzyme system, both G3A and CatG4 were effectively cleaved by the activated Cas12a in the presence of the target sequence (Fig. 3D). Visual assessment confirmed that CatG4 provided the most distinct color contrast between positive and negative samples. Based on these results, CatG4 was selected as the reporter molecule for the visual detection system.

### Sensitivity and specificity evaluation of the detection methods

**Fig 4:**
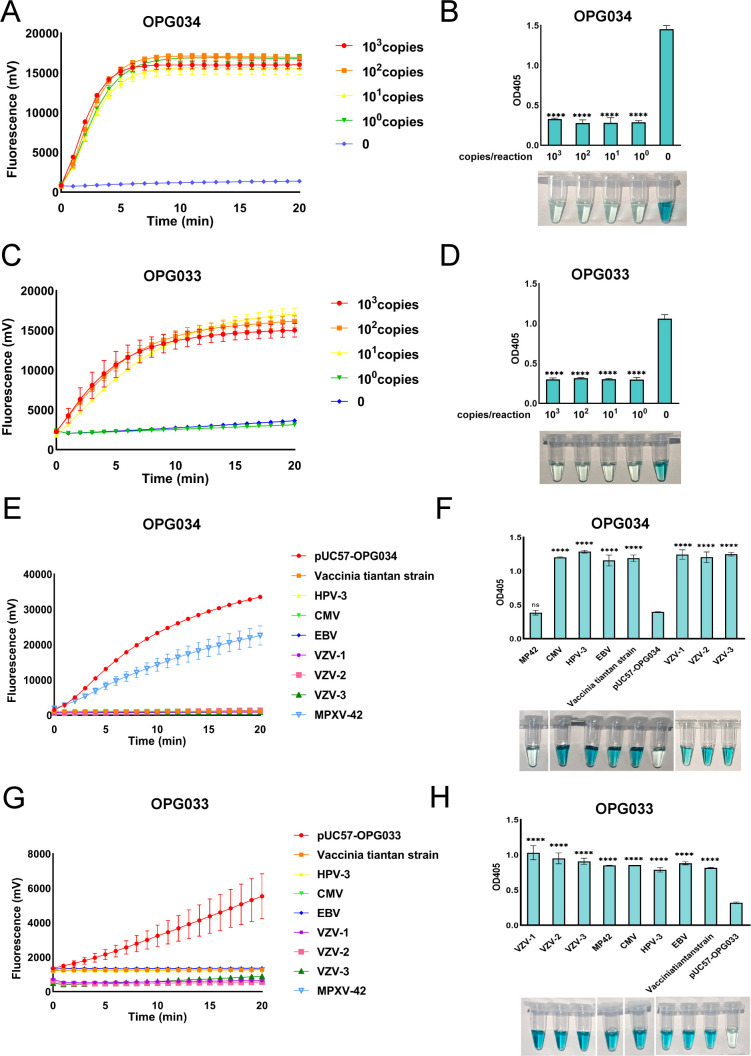
Sensitivity and specificity evaluation of the DUAL-CRISPR-MPXV detection system. (**A–D**) Limit of detection assessment using gradient-diluted pUC57-OPG034 (**A and B**) and pUC57-OPG033 (**C and D**) plasmids as templates. (**A and C**) Fluorescence readout; (**B and D**) visual readout. (**E–H**) Specificity evaluation: (**E and F**) detection results for the MPXV universal target (OPG034); (**G and H**) detection results for the MPXV Clade I-specific target (OPG033). (**E and G**) Fluorescence method; (**F and H**) visual method. All data are presented as mean ± SD from two technical replicates. One-way ANOVA with Dunnett’s multiple comparison test was applied to analyze intergroup differences in panels **B, D, F, and H**. Significance levels: ns, not significant; ****, *P* < 0.0001.

To systematically evaluate the detection performance, we performed sensitivity testing using serially diluted pUC57-OPG034 and pUC57-OPG033 plasmids (1–1,000 copies/μL) as templates. For OPG034 detection, both the fluorescence and visual detection methods exhibited a LOD of 1 copy/reaction (Fig. 4A and B). In the visual detection system, only the negative control displayed color development, confirming the detection sensitivity. Regarding OPG033 detection, the fluorescence method showed an LOD of 10 copies/reaction, whereas the visual method achieved 1 copy/reaction (Fig. 4C and D), indicating higher sensitivity of the visual method for OPG033 detection.

The specificity was assessed using various clinically validated samples, including one vaccinia virus tiantan strain, three VZV lesion fluid samples, one EBV serum sample, one CMV serum sample, one HPV-3 serum sample, and one confirmed MPXV Clade II-positive sample. The results demonstrated that in OPG034-specific detection, both fluorescence and visual methods specifically identified MPXV targets without cross-reactivity with other viruses (Fig. 4E and F). In OPG033-specific detection, only the pUC57-OPG033 plasmid produced a positive signal, while the MPXV Clade II sample and all other viral samples tested negative (Fig. 4G and H), which was fully consistent with expectations. These findings confirm that the established universal and Clade I-specific detection methods for MPXV possess excellent specificity.

### Detection performance of DUAL-CRISPR-MPXV for MPXV in clinical samples

To evaluate the detection efficacy of the DUAL-CRISPR-MPXV system in real clinical settings, we systematically analyzed 23 clinical samples from MPXV-infected individuals and 10 healthy controls. After prolonged sample storage at −80°C, all samples were retested by qPCR. Using a Ct ≤37 cutoff as the reference standard in our comparative analysis, 13 samples were classified as qPCR-positive, while 10 samples (originating from confirmed patients but with Ct >37 or undetermined upon retesting) were classified as qPCR-negative (Table S1).

**Fig 5:**
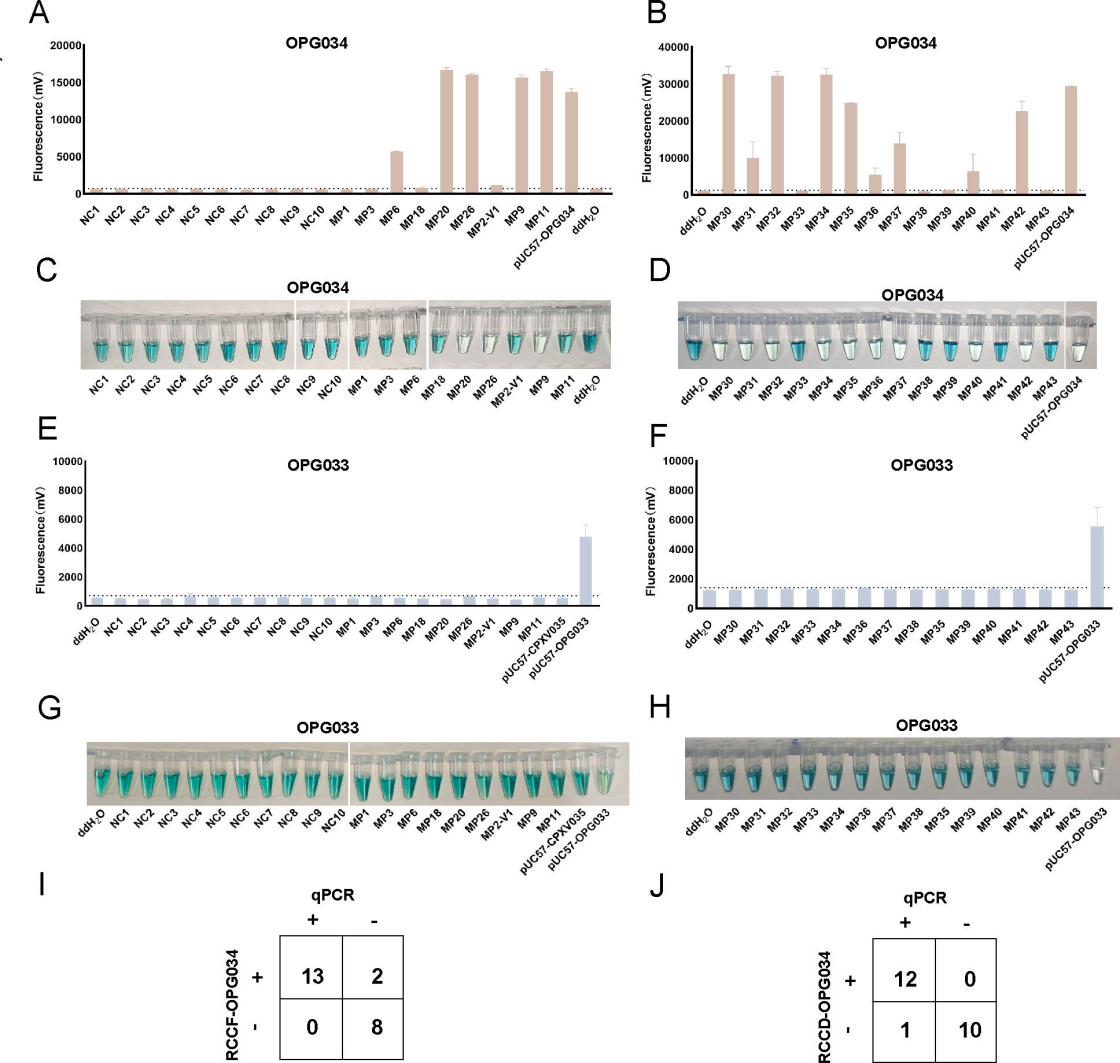
Detection of MPXV in clinical samples using the DUAL-CRISPR-MPXV system. (**A–D**) Detection based on the OPG034 universal target: (**A and B**) fluorescence readout; (**C and D**) visual readout. (**E–H**) Detection based on the OPG033 clade-specific target: (**E and F**) fluorescence readout; (**G and H**) visual readout. (**I**) Correlation between qPCR and RCCF-OPG034 results for mpox samples. (**J**) Correlation between qPCR and RCCD-OPG034 results for mpox samples. MP, MPXV-infected patient samples (*n* = 23); NC, healthy control samples (*n* = 10). All experiments were performed in duplicate. The dashed black line indicates the positivity threshold, defined as the mean of the negative control (ddH_2_O) plus three standard deviations.

Using the qPCR results (Ct ≤37 cutoff) as the reference standard, we calculated the sensitivity and specificity of each assay with 95% confidence intervals (Wilson score method). For universal detection targeting OPG034, the fluorescence method (RCCF-OPG034) detected 13 samples that were qPCR-positive and 2 additional samples that were qPCR-negative, yielding a sensitivity of 100% (13/13, 95% CI: 77.2%–100%) and a specificity of 80.0% (8/10, 95% CI: 49.0%–96.5%) (Fig. 5A and B). The visual method (RCCD-OPG034) detected 12 of the 13 qPCR-positive samples and none of the qPCR-negative samples, corresponding to a sensitivity of 92.3% (12/13, 95% CI: 66.7%–99.6%) and a specificity of 100% (10/10, 95% CI: 72.2%–100%) (Fig. 5C and D).

When compared directly with qPCR results, the CRISPR fluorescence method showed complete concordance (100%) with qPCR for samples with Ct values ≤ 37. Notably, two samples that tested negative by qPCR (Ct >37) were positive by the CRISPR fluorescence method. Given that our assay targets a different genomic region (OPG034) than the qPCR assay, this discrepancy may reflect differential preservation or distribution of target sequences in low-copy-number samples. The visual method detected one fewer positive sample (12 samples) than qPCR, yielding a positive agreement rate of 92.3% (12/13) with the qPCR results. The undetected sample had a qPCR Ct value of 36.465, indicating a relatively low viral load. This observation can be attributed to the design of the visual method as an instrument-free, naked-eye readout system. At extremely low viral loads (Ct >36), the color change becomes minimally distinguishable from the negative control, reflecting an inherent sensitivity limitation of visual detection under low-target conditions.

For the Clade I-specific assays targeting OPG033, both fluorescence (RCCF-OPG033) and visual (RCCD-OPG033) methods demonstrated 100% specificity for Clade I detection in the 23 Clade II samples (23/23, 95% CI: 85.7%–100%) (Fig. 5G through J). The sensitivity for Clade I detection could not be assessed due to the absence of authentic Clade I clinical specimens. It should be noted that although MPXV Clade IIb is the predominant strain circulating in China, and Clade I samples were unavailable during this study (China first reported Clade Ib cases in January 2025), all 23 samples tested negative for the Clade I-specific OPG033 target. This result is consistent with the fact that all samples belonged to Clade II, confirming the specificity and reliability of the DUAL-CRISPR-MPXV system for clade-specific identification in a clinical context.

## DISCUSSION

In this study, we performed genomic alignment of MPXV using MAUVE and identified two novel MPXV-specific gene sequences: OPG034 and OPG033. OPG034 serves as a universal detection target conserved in both clades. Although this sequence is also present in CPV, we achieved specific MPXV detection by designing crRNA targeting a region with nucleotide variations. While OPG033 shows high homology with CPV and CMLV, we eliminated cross-reactivity through a crRNA mismatch strategy, enabling specific identification of MPXV Clade I.

Current research on MPXV clade identification remains limited, with most reported methods focusing on universal detection. Chen et al. established a LAMP-CRISPR/Cas12b detection system based on *D14L* and *ATI* genes capable of distinguishing different clades, but its validation relied solely on simulated samples without clinical sample support (19). Zhang et al. reported a real-time PCR method that can differentiate between Clade I and Clade II within 40 min (17–22), but its dependence on specialized instrumentation limits its application in field detection. In contrast, the dual-mode CRISPR-Cas12a platform established in our study not only provides high sensitivity but also enables visual readout without requiring professional equipment, making it suitable for rapid screening in resource-limited settings. A comparative summary of our platform with previously reported CRISPR-based MPXV detection methods is presented in Table 2, highlighting key features including LOD, assay time, workflow complexity, clade typing capability, equipment needs, and clinical validation.

**TABLE 2:** Comparison of CRISPR-based MPXV detection platforms

	This study—fluorescence mode	This study—visual mode	Chen et al. (28)	Zhao et al. (26)	Hu et al. (29)	Guo et al. (20)	Chen et al. (19)
Target gene	OPG034/OPG033	OPG034/OPG033	A27L	F3L	F3L	F3L	D14L/ATI
LOD	1 copy/reaction	1 copy/reaction	10 aM (~6 copies)	10 copies/μL	4 copies	6.5 copies	10 copies
Assay time	55 min	95 min	40 min	70 min	50 min	40 min	60 min
Workflow complexity	Two-step RAA-CRISPR/Cas12a	Two-step RAA-CRISPR/Cas12a-DNAzyme	Single-step RPA-CRISPR/Cas12a	Two-step RAA-CRISPR/Cas12a	Single-tube two-step MIRA-CRISPR/Cas12b	One-pot LAMP-CRISPR/Cas12b	Two-step LAMP-CRISPR/Cas12b
Clade typing	Yes (OPG033)	Yes (OPG033)	No	No	No	No	Yes
Equipment needs	Fluorescence reader	None (visual)	Fluorescence reader or LFA strips	Fluorescence reader or LFA strips	Fluorescence reader or blue light	Blue/UV light	Fluorescence reader or AuNP-LFB
Clinical validation	23 Clade II samples	23 Clade II samples	30 samples (six types)	None (plasmids only)	100 samples	113 samples	Simulated samples (*n* = 60)

We constructed a “dual-CRISPR-MPXV” platform supporting both fluorescence and visual readouts. Unlike traditional CRISPR methods relying solely on fluorescence (40, 41), our system meets the requirements for both high-sensitivity laboratory testing and field screening, significantly enhancing its practicality and application scope.

All clinical samples used in this study were collected from patients at various stages of infection. In some cases, patients presented with mild symptoms at the time of collection, suggesting they may have been in the recovery period when viral loads are typically low. Additionally, all samples experienced prolonged storage at −80°C for over 1 year as plasma and swab specimens prior to our analysis. Studies have shown that even under optimal freezing conditions, long-term storage can lead to gradual nucleic acid degradation. When we retested all samples after prolonged storage using a standardized qPCR assay with a Ct ≤ 37 cutoff, only 13 of the 23 samples were classified as qPCR-positive, while 10 samples (originating from confirmed patients but with Ct >37 or undetermined upon retesting) were classified as qPCR-negative. If we were to use the original clinical diagnosis (established at the time of patient presentation, when viral loads were at their peak) as the reference standard, the calculated sensitivity of our assays would be artificially lowered due to sample quality deterioration, not due to inherent assay deficiencies. This is evident from the similarly reduced sensitivity of qPCR itself (56.5%) when referenced against the original diagnosis.

Based on this rationale, we evaluated our assays against the retest qPCR results. During clinical validation, our method detected two additional positive samples (MP11 and MP2-V1) that were classified as negative by qPCR (Ct >37). These samples were collected from confirmed MPXV-infected patients but had relatively high Ct values upon initial testing. Given that our assay targets a different genomic region (OPG034) than the qPCR assay, the observed discrepancy may reflect differential preservation or distribution of target sequences in low-copy-number samples. As such, our method could serve as a complementary approach for detecting low-viral-load specimens, though confirmatory testing (e.g., digital PCR) would provide additional confidence.

The OPG034 visual method showed excellent agreement with qPCR (positive agreement rate 92.3%), with the single discordant sample having a Ct value of 36.465. This reflects the inherent sensitivity limitations of instrument-free visual detection under extremely low target conditions. This trade-off between portability and sensitivity is acceptable given the visual method’s intended use for on-site rapid screening rather than quantitative analysis.

Beyond analytical performance, the practical utility of our platform for point-of-care testing is reflected in key operational metrics summarized in Table 3, which provides a direct comparison with conventional qPCR. Notably, the visual detection mode enables instrument-free operation with a total turnaround time of approximately 95–100 min, a reagent cost of $2.3–$2.5 USD per sample (significantly lower than qPCR), and minimal user training (1–2 h). Reagents can be stored at 2°C–8°C, with lyophilized components reconstituted before use. While sample preparation currently requires a commercial extraction kit, future efforts will explore simpler methods such as DNA release agents to further enhance field applicability. Additionally, by redesigning primers and crRNAs, this platform can be readily adapted for detecting other pathogens, demonstrating excellent versatility and expandability.

**TABLE 3:** Comparison of key operational metrics for field deployment

Metric	Our platform (visual mode)	Conventional qPCR
Total turnaround time	~95–100 min (extraction 15–20 min + RAA 20 min + detection 60 min)	~2–3 h
Estimated cost per test	$2.3–$2.5 USD	$5–$10 USD
Instrument requirement	Simple constant-temperature device, such as a thermos flask	Thermal cycler + fluorescence detector
Reagent storage	2°C–8°C (RAA kit, Cas12a, detection kit); lyophilized primers/crRNA stable at room temperature	−20°C or −80°C
Sample preparation	Commercial extraction kit required	Commercial extraction kit required
User training	Minimal (1–2 h)	Specialized training required
Contamination-control strategies	Physical separation of pre-/post-amplification areas; aerosol-resistant tips; future one-pot closed-tube system needed	Closed-tube system; built-in contamination controls

Despite these promising features, this study has several limitations. All clinical samples were obtained from MPXV Clade II cases during 2023–2024, and thus the typing performance of OPG033 requires further validation with authentic Clade I clinical specimens. The two-step RAA-CRISPR workflow carries a risk of amplicon contamination due to tube opening. Although standard precautions (e.g., physical separation of work areas, aerosol-resistant tips, and negative controls) are recommended to mitigate this risk, future efforts will focus on developing a one-pot closed-tube system to eliminate contamination entirely, as reported in recent studies (18, 19). Additionally, while our assays demonstrated 100% specificity, the sensitivity values observed were modest due to sample quality limitations; future studies using fresh clinical specimens would provide a more accurate assessment of assay performance.

### Conclusions

In summary, we developed a dual-mode detection platform by integrating RAA pre-amplification with a CRISPR-Cas12a system. Utilizing both fluorescence and visual readouts, this approach enables sensitive, universal detection and clade identification of MPXV, validated with high specificity and sensitivity in clinical samples. The platform also demonstrates proof-of-principle for clade discrimination based on target sequence selection, providing a versatile tool for MPXV screening. However, validation with authentic Clade I clinical specimens is required to confirm the discriminatory performance of the OPG033 assay.

## SUPPLEMENTAL MATERIAL

Supplemental material spectrum.00105-26-s0001.docx Fig. S1 to S9; Table S1.

ASM does not own the copyrights to Supplemental Material that may be linked to, or accessed through, an article. The authors have granted ASM a non-exclusive, world-wide license to publish the Supplemental Material files. Please contact the corresponding author directly for reuse.
