# Supplementary material for: CRISPR-Cas12a-based fluorescent and visual assays for universal detection and clade discrimination of mpox virus
Source: Microbiol Spectr. 2026 Jun 22;14(8):e00105-26. doi: 10.1128/spectrum.00105-26 (PMC13435695; doi:10.1128/spectrum.00105-26)
Supplement: Supplementary file 1 [file spectrum.00105-26-s0001.docx]

**Supporting information**

**Figure S1. Nextclade phylogenetic analysis interface for the OPG034 sequence, showing the initialization screen with the selected reference set "Mpox virus (All clades)".**

**Figure S2. Phylogenetic tree of the OPG034 sequence constructed using Nextclade.**

**Figure S3. BLAST alignment results of the OPG034 sequence against monkeypox virus genomes.**

**Figure S4. BLAST alignment results of the OPG034 sequence against non‑monkeypox virus genomes.**

**Figure S5. Nucleotide alignment of the OPG034 sequence with Cowpox virus strain Austria 1999.**

**Figure S6. Nextclade phylogenetic analysis interface for the OPG033 sequence, showing the initialization screen with the selected reference set "Mpox virus (Clade I)".**

**Figure S7. Phylogenetic tree of the OPG033 sequence constructed using Nextclade.**

**Figure S8. BLAST alignment results of the OPG033 sequence against monkeypox virus genomes.**

**Figure S9. Nucleotide alignment of the OPG033 sequence with Cowpox virus strain CPXV/Cepad 333.**

**Table S1. Comparison of qPCR and DUAL-CRISPR-MPXV for Detection of MPXV in 33 Clinical Samples.**

| Sample ID | qPCR (Ct value) | qPCR | RCCF-OPG034 | RCCD-OPG034 | RCCF-OPG033 | RCCD-OPG033 |
| --- | --- | --- | --- | --- | --- | --- |
| MP1 | 37.823 |  |  |  |  |  |
| MP3 | 39.545 |  |  |  |  |  |
| MP6 | 36.465 | + | + |  |  |  |
| MP11 | ND |  | + |  |  |  |
| MP18 | 37.946 |  |  |  |  |  |
| MP20 | 29.097 | + | + | + |  |  |
| MP26 | 27.634 | + | + | + |  |  |
| MP2-V1 | 37.547 |  | + |  |  |  |
| MP9 | 13.495 | + | + | + |  |  |
| MP30 | 27.114 | + | + | + |  |  |
| MP31 | 30.291 | + | + | + |  |  |
| MP32 | 28.678 | + | + | + |  |  |
| MP33 | 38.841 |  |  |  |  |  |
| MP34 | 28.977 | + | + | + |  |  |
| MP35 | 29.338 | + | + | + |  |  |
| MP36 | 33.691 | + | + | + |  |  |
| MP37 | 29.742 | + | + | + |  |  |
| MP38 | ND |  |  |  |  |  |
| MP39 | ND |  |  |  |  |  |
| MP40 | 32.008 | + | + | + |  |  |
| MP41 | ND |  |  |  |  |  |
| MP42 | 24.756 | + | + | + |  |  |
| MP43 | ND |  |  |  |  |  |
| NC1 | ND |  |  |  |  |  |
| NC2 | ND |  |  |  |  |  |
| NC3 | ND |  |  |  |  |  |
| NC4 | ND |  |  |  |  |  |
| NC5 | ND |  |  |  |  |  |
| NC6 | ND |  |  |  |  |  |
| NC7 | ND |  |  |  |  |  |
| NC8 | ND |  |  |  |  |  |
| NC9 | ND |  |  |  |  |  |
| NC10 | ND |  |  |  |  |  |

"+" denotes positive samples; n = 33 represents the total sample size.
